# Virtual surgical education for core surgical trainees in the Yorkshire deanery during the COVID-19 pandemic

**DOI:** 10.1177/0036933020951927

**Published:** 2020-09-02

**Authors:** Ryan Laloo, Andrea Giorga, Andrew Williams, Chandra Shekhar Biyani, Marina Yiasemidou

**Affiliations:** 1 Academic Clinical Fellow in Vascular Surgery, Leeds Teaching Hospitals Trust, UK; 2 General Surgery ST2, Leeds Teaching Hospitals Trust, UK; 3 Consultant Plastic Surgeon, Bradford Teaching Hospitals NHS Foundation Trust, UK; 4 Consultant Urologist, Leeds Teaching Hospitals Trust, UK; 5 General Surgery registrar, Leeds Teaching Hospitals Trust, UK *Ryan Laloo and Andrea Giorga were both first co-authors.

**Keywords:** COVID-19, surgical education, surgical training

## Abstract

**Background and Aims:**

An online teaching programme for Core Surgical Trainees (CSTs) was designed and delivered during the COVID-19 pandemic. The aim of this study is to assess the feasibility and the reception of a fully online teaching programme.

**Methods:**

Twenty teaching sessions were delivered either via *Zoom™* or were pre-recorded and uploaded onto a *Google Classroom™* and *YouTube™* website. Online feedback, delivered via *Google Forms™*, were completed by CSTs following each teaching session. *YouTube Studio™* analytics were used to understand patterns in viewing content.

**Results:**

89.9% of trainees were satisfied with the teaching series. Trainees preferred short, weekly sessions (79%), delivered by senior surgeons, in the form of both didactical and interactive teaching. YouTube analytics revealed that the highest peak in views was documented on the weekend before the deadline for evidence upload on the Intercollegiate Surgical Collegiate Programme (ISCP) portfolio.

**Conclusion:**

An entirely online teaching programme is feasible and well-received by CSTs. Trainees preferred live, interactive, procedure-based, consultant-led sessions lasting approximately thirty minutes to one hour and covering a myriad of surgical specialties. This feedback can be used to improve future online surgical teaching regionally and nationally in order to gain training opportunities lost during the pandemic.

## Background

The World Health Organisation declared COVID-19 a pandemic on March 11th 2020 causing significant alterations to most aspects of personal and professional life.^1^,^2^ On March 23rd 2020, the United Kingdom (UK) government implemented social distancing rules requiring people to stay at home except for very limited purposes, close certain businesses and stop all gatherings of more than two people in public.^3^ Consequently, surgical training was negatively affected nationwide with the suspension of non-urgent elective surgery and reduction in face-to-face lectures, practical teaching sessions and dedicated operative theatre time.^4^

Two core surgical trainees (CSTs) alongside the Training Programme Director from the Yorkshire & Humber deanery in the UK designed an online teaching programme aimed at CSTs within the deanery. This was delivered by supervised CSTs, surgical registrars and consultants on a twice-weekly basis commencing April 22nd 2020. A consultant surgeon was allocated as supervising faculty for most teaching sessions. The purpose of the teaching programme was to promote high quality surgical education and facilitate on-going learning while maintaining engagement with the Intercollegiate Surgical Collegiate Programme (ISCP) portfolio.^5^

The primary aim of this study was to assess the feasibility of an entirely on-line teaching programme for CSTs. The secondary aims included (i) assessing the reception of the novel teaching method by trainees and (ii) identifying training needs in order to allow organisers to adapt accordingly when designing future sessions.

## Methods

Twenty online teaching sessions were delivered from the commencement of the programme on April 22nd 2020 to June 12th 2020. Ten sessions were led by CSTs and ten were led by senior surgical registrars and consultant surgeons. Twice-weekly sessions, each lasting between thirty minutes to one hour, were either recorded live or pre-recorded and delivered online using *Zoom™ (Zoom Video Communications, San Jose, CA)*. The form of the teaching session (pre-recorded or live) was left in the speaker’s discretion based on how they thought the content was best delivered.

As the study involved trainees and not patients, ethical approval was not necessary. Consent for recording and dissemination of content was obtained from all tutors and participants, in addition to compliance with the ‘Recording of Webinars and other teaching sessions’ policy as suggested by Health Education England (HEE).^6^ A moderator was assigned at the beginning of each session to chair an interactive ‘question and answer’ segment which consolidated learning at the end of each session. The recordings were quality controlled and approved by the surgical Training Programme Director before being uploaded to the Yorkshire and Humber Deanery’s *YouTube™* channel for future access. Video recordings were available to surgical trainees using a web link and not the general public. A *Google Classroom™ website* was created with web links to the video recordings, online feedback forms and regular updates on upcoming teaching sessions. Questionnaires, and all data extracted from them was anonymised, and analysed according to themes and not used verbatim.

The teaching content was in accordance with the Joint Committee of Surgical Training (JCST) curriculum and Membership of the Royal College of Surgeons (MRCS) examinations.^7^ CSTs selected topics from an approved list generated from the Yorkshire and Humber School of Surgery and prepared a *Microsoft PowerPoint™* slide presentation with supervision from a senior surgical registrar or consultant surgeon in the respective specialty.

Sessions delivered by senior surgical registrars and consultant addressed specific intra-operative principles, common pitfalls and strategies to reduce intra and post-operative complications. These ‘Procedure-based’ sessions included procedures such as inguinal hernia repair, laparoscopic cholecystectomy and examination under anaesthesia of the ano-rectum. Sub-specialty topics (e.g. paediatric surgery) were most often delivered by consultant surgeons.

The number of attendants varied from 15 to 30 trainees for each live session. Although no formal attendance was recorded, CSTs were encouraged by the Training Programme Director to document their reflections on the key learning points from teaching sessions on their ISCP portfolio.

Feedback on the sessions was collected using online questionnaires (created using *Google Forms*™) which could be found on the *Google Classroom™ website.* Trainees were notified via Deanery wide *WhatsApp™* group chats. The completion of the feedback form was voluntary, and anonymised. An additional Google form on the Overall Teaching Series was generated and disseminated in *Google Classroom™* and via *WhatsApp™* group chats. *Google Forms™* were anonymously filled by CSTs of the Yorkshire & Humber region and were available to be completed for the entire duration of the programme. Results were automatically summarised, and available to the teaching organisers immediately after the end of every session. The use of the summarised, anonymised data for publication purposes was explained to the trainees via email, in such a manner the trainees were given the opportunity to express their objection to this if they wished.

In addition, the use of *YouTube*™ Studio Analytics was employed on the Deanery’s *YouTube™* channel in order to provide information on the number of subscribers and views over time.

## Results

Twenty sessions were delivered over the seven week programme, by the end of which 62 CSTs were subscribed to *Google Classroom™* and 46 to the *YouTube™* channel. The teaching programme included a wide range of surgical specialties as detailed in Table 1 including General Surgery (7), Plastic Surgery (3), Vascular Surgery (2), Trauma & Orthopaedics (2), Paediatric Surgery (1), Urology (1), Oral & Maxillofacial Surgery (1) and others (3).

**Table 1.** Outline of online surgical teaching programme.

Topic	Type	Speaker
Vascular surgery	Live	CST
Gastrointestinal bleeding	Live	CST
Working as a registrar	Live	Registrar
Plastic surgery – an overview	Pre-recorded	Consultant
Renal replacement therapy and vascular surgery	Live	CST
Abdominal Pain - An Approach	Pre-recorded	Registrar
Care of the critically ill surgical patient	Pre-recorded	Registrar
Free flap monitoring	Pre-recorded	CST
Expert topic: pancreatic surgery	Live	Post CCT Fellow
Leadership, team management & professional communication	Live	Post CCT Fellow
Benign and malignant bone disease	Live	CST
Surgical principles: inguinal hernias	Live	Post CCT Fellow
Neck lumps	Pre-recorded	CST
Gynaecological causes of abdominal pain	Live	CST
Prostate cancer	Pre-recorded	CST
Burns: an overview	Pre-recorded	CST
Hand trauma	Pre-recorded	CST
Expert topic: paediatric surgery	Live	Consultant
Surgical principles: EUA of the anorectum	Live	Consultant
Surgical principles: laparoscopic cholecystectomy	Live	Consultant

Post CCT Fellow: Post Certificate of Completion of Training (CCT) fellow; EUA: examination under anaesthesia.

**Figure 1.**
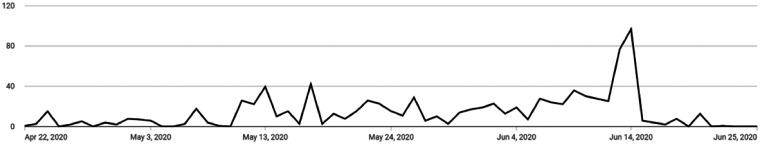
YouTube channel analytics displaying the total number of views over time.

*YouTube Studio™* analytics data indicated that by the end of the programme there were 46 channel subscribers, 1100 views and 108.3 hours of viewing time in total. There were on average 6.3 views per viewer and the highest number of views (19% of total views) was recorded on the 13th & 14th June, the weekend before ISCP portfolio deadline for evidence submission (23:59 on 14th of June). The number of views over time is displayed in Figure 1. A surge of views was observed a couple of days after the upload of a new recording and on weekends. Data on the most viewed videos (Table 2) was available to the series organisers, and was used to shape the programme accordingly. Trainees used various devices for accessing the YouTube channel with 59.9% using a computer, 30.6% a mobile phone, 7.1% a game console, 2.3% a tablet and 0.1% TV.

**Table 2.** Five most viewed videos on the YouTube channel, over the programme teaching duration.

	Views	Views (% of Total)	Watch Time (hours)	Watch time (% of Total)
Total	1103		108.5	
Gastrointestinal Bleeding	132	12.0%	8.8	8.1%
Plastic surgery (part 1)	120	10.9%	7	6.5%
Vascular teaching	100	9.1%	10.8	9.9%
Plastic surgery (part 2)	75	6.8%	5.6	5.2%
Life as a registrar	63	5.7%	9.5	8.8%

Online feedback forms were voluntary as their completion was not used to record attendance. The 64 trainees subscribed to *Google Classroom™* were reminded about feedback forms monthly. A total of 160 online feedback forms were completed by CSTs for the 20 teaching sessions including 146 responses for individual sessions and 14 responses for the overall teaching programme (Questionnaires 1 and 2 respectively). Since completion of feedback form was voluntary and attendance was not formally recorded, the response rate was not calculated.

**Figure 2.**
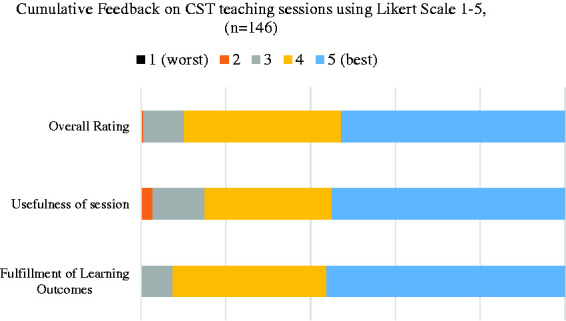
Cumulative feedback results from individual sessions (Questionnaire 1).

Feedback data on individual sessions (Figure 2) indicated that 90% trainees rated the sessions highly, 85% found each session very useful and 92% indicated that each session fulfilled the learning objectives (4 & 5 on a Likert scale of 1 to 5 – 1: not satisfied at all, 5: very satisfied).

**Figure 3.**
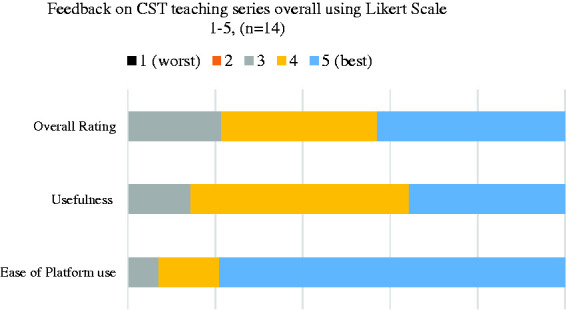
Cumulative feedback results from the overall teaching programme (Questionnaire 2).

Data from trainee feedback on the series as a whole (Figure 3) revealed that 79% of trainees rated the teaching series highly, and 86% found of the teaching series useful (4 & 5 on a Likert scale of 1 to 5 – 1: not satisfied at all, 5: very satisfied). 93% of trainees were very satisfied with the platforms used (Google Classroom™, Google Forms™ and the Yorkshire and Humber deanery YouTube™ channel).

**Figure 4.**
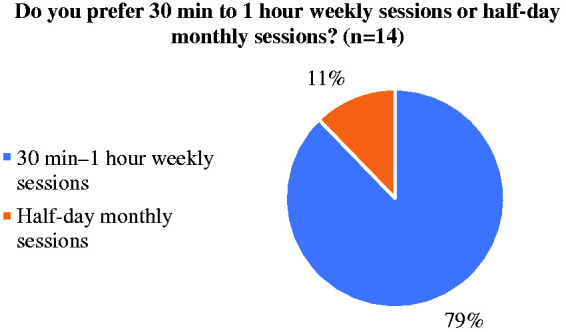
Trainees preferences regarding duration and frequency of online teaching sessions.

Most of the trainees preferred a weekly 30 minute to an hour online session as compared to a half-day monthly session (Figure 4).

Thematic analysis was conducted for the ‘free text’ data from both questionnaires. The emerging themes were:

1. **Later start time.** Even though recorded sessions had more views over weekends (Figure 1), trainees did not express any strong preferences on which day of the week the live sessions should take place. However, 30% preferred a later start time, later than 18:00, during the week.
2. **Procedure-based and Consultant-led teaching.** Trainees expressed a strong preference for ‘Procedure-based’ teaching sessions. They also requested more consultant-led teaching sessions.
3. **Wider range of surgical specialties.** Although the teaching programme (Table 1) included topics from a myriad of surgical specialties and mostly fulfilled the learning outcomes (Figure 2), the majority of the sessions were on General Surgery and Plastic Surgery (50%). Trainees requested more sessions on other specialties, as represented in the Membership of the Royal College of Surgeons (MRCS) syllabus.
4. **Use of online platforms.** The use of the aforementioned platforms was not only widely accepted as an alternative to traditional monthly group face-to-face sessions during the COVID19 pandemic, but also offered a variety of advantages over them: a) Recordings of sessions: Some trainees stated that working commitments occasionally restricted their ability to attend live teaching sessions but benefitted from reviewing the recorded videos at a time of their convenience. b) Time-efficiency: Approximately 30% of trainees expressed a strong preference for receiving didactic lectures via online platforms as a means of reducing unnecessary travel across cities even after the pandemic restrictions are uplifted. In terms of the quality & accessibility of the recordings all trainee responses were positive, apart from one which indicated minor issues.
5. **Continued need for practical sessions.** Trainees have stated that once the government’s social distancing guidance permits, they would be keen on resuming the usual practical skills sessions.

**Table:** 

Questionnaire 1. Individual Session feedback form	
Q1	To what extent did the session fulfil the learning outcomes?
Q2	How useful/relevant was the session to your role and learning needs?
Q3	How would you rate the teaching session overall?
Q4	How will this change/reinforce your practice going forward?
Q5	Additional comments?
Questionnaire 2. Overall teaching programme feedback form	
Q1	How useful/relevant are the sessions to your role and learning needs?
Q2	Overall how would you rate the teaching sessions so far?
Q3	What are your thoughts on the day, time and duration of the teaching sessions delivered?
Q4	Do you find having short sessions every week suitable or would you prefer multiple sessions in one day a month?
Q5	What are your thoughts on the quality & accessibility of the recordings of the sessions?
Q6	How easy to use do you find the current platforms? (Zoom/YouTube/Classroom)
Q7	Additional comments and suggestions on the Platforms/Format
Q8	Other suggestions or comments

## Discussion

An entirely online teaching programme was feasible and well received by CSTs in the deanery of Yorkshire, during the COVID-19 pandemic. The findings of this survey are consistent with findings in previous educational studies.

Current literature indicates that shorter interactive sessions can promote information retention and attentiveness of the trainees since adult attention spans can wane after 15 to 20 minutes and hence, Hopkins et al stated that millennials favour interactive shorter case discussions instead of a lecture in a ‘flipped classroom’ format.^8^ This involves teamwork, debates, application and consolidation to promote a highly level of understanding and information retention.^8^

Online teaching can be aided by the use of multimedia resources such as anatomy diagrams, intraoperative pictures and videos, in order to enhance learning.^9^ As traditionally known, persons retain only 10–15% of what is read, 10-20% of what is heard and 20–30% of what is seen. However, retention increases to 40–50% with the combined use of audio-visual supplements.^10^ In fact, Crawshaw et al stated that an instructional video before performing surgery may reduce the learning curve of trainees and improve patient safety.^11^

Gilbert et al highlighted the benefit of educational websites to facilitate unlimited distribution of information to students using web links with the benefits of convenience, efficiency in learning, unlimited viewing, and the ability to access physical distance.^12^ Hands-on simulation training is paramount in surgical education and this would form a vital part of resumption to usual trainee skill acquisition. Kolsanov et al highlighted that simulation offers outstanding learning opportunities in a safe environment without negative patient outcomes.^13^

## Limitations

This study has some limitations. The teaching programme was voluntary, aimed to facilitate learning during the pandemic and enable CSTs to deliver teaching sessions as required by the ISCP curriculum. However, reflecting on teaching sessions on the ISCP portfolio as encouraged by the Deanery, appeared to be the major motivating factor as discussed above. This resulted in a low survey response rate. Going forward, the recording of attendance might increase trainee engagement with the programme as well as improve the survey response rate. In addition, issuing certificates of attendance could improve engagement.

Another challenge was the unavailability of more experienced senior surgeons to deliver teaching sessions particularly during the peak of the pandemic. In the future, we aim to engage more senior speakers through the involvement of Royal College of Surgeons college tutors, who possess a wealth of expertise on various surgical specialties to further enhance the quality and relevance of the teaching sessions.

## Recommendations

Taking into consideration the results of this survey, the existing literature on the subject as well as the challenges faced during the delivery of the teaching series, we have some recommendations:

Online teaching for CSTs should preferably consist of short, weekly sessions delivered at a convenient time but also be recorded and available for viewing at a later date. There should be high consultant involvement, from a range of surgical specialties. Teaching sessions should be interactive, procedure-based and exam-focused. The utilisation of online platforms, universally compatible with all devices, should aid tailoring of the teaching programme to trainees’ learning needs. In addition, incentives in the form of completion certificates and attendance records, can increase trainee engagement. Finally, designated practical skills sessions should continue to be incorporated in the curriculum and the use of simulation models can facilitate this further in the future.

Our team of Yorkshire surgical trainees intend on using this valuable trainee feedback to improve and influence our regional upcoming online teaching sessions and provide suggestions to CSTs nationwide in order to maximise training opportunities lost during the pandemic.

## Conclusion

A multispecialty fully-online teaching programme for core surgical trainees can be feasible and well-received. This study provides insight to improve future online teaching programmes according to the educational needs of trainees.
